# Platform-specific impact of CT attenuation correction on CZT myocardial perfusion SPECT: per-vessel detection of obstructive coronary artery disease

**DOI:** 10.1007/s10554-026-03791-w

**Published:** 2026-08-12

**Authors:** Yoshimitsu Honda, Yoshimitsu Fukushima, Kenji Yodogawa, Yujiro Nakaoka, Hiromitsu Hayashi, Shinichiro Kumita

**Affiliations:** 1 Department of Radiology, Nippon Medical School, Tokyo, Japan; 2 Department of Cardiovascular Medicine, Nippon Medical School, Tokyo, Japan

**Keywords:** Myocardial perfusion imaging, Attenuation correction, Cadmium–zinc–telluride SPECT, Coronary artery disease, Hybrid cardiac SPECT/CT imaging, Diagnostic accuracy

## Abstract

Attenuation artifacts on myocardial perfusion SPECT differ across camera geometries. This study evaluated whether CT attenuation correction (CTAC) improves per-vessel detection of obstructive coronary artery disease (CAD) on a stationary 19-pinhole CZT system. Rest–stress ⁹⁹ᵐTc-tetrofosmin CZT SPECT studies from 48 patients with angiographically confirmed CAD (185 vessels) were retrospectively reconstructed with and without CTAC. The reference standard was ≥ 70% diameter stenosis on coronary CT angiography or invasive angiography, with no perfusion input. Disease-negative participants were excluded by design. Perfusion was quantified as the percent defect averaged within each coronary territory, assigned per patient on hybrid SPECT/CT fusion images. Per-vessel discrimination of the reversible (stress − rest) defect rate was compared between reconstructions. Compared with non-attenuation-corrected (NAC) reconstructions, CTAC lowered the global rest defect rate (*P* < 0.0001) and raised the reversible defect rate (median 5.9% vs. 1.5%; *P* < 0.0001). The reversible defect rate discriminated territories subtended by obstructive stenosis better with CTAC than with NAC (AUC 0.717 vs. 0.614; ΔAUC 0.103; DeLong *P* = 0.0164; bootstrap *P* = 0.010; within-patient permutation *P* = 0.0474). At the Youden point, sensitivity rose to 75.5% from 52.8%, with specificity 64.4% versus 68.9%. The gain was greatest in the LCX (0.803 vs. 0.658; *P* = 0.043) and RCA territories. On this 19-pinhole CZT platform, CTAC improves per-vessel discrimination of obstructive CAD, most prominently in the LCX and RCA territories. The benefit is sensitivity-led, opposite to the specificity-led gain reported on Anger cameras, at a modest cost in specificity.

**Graphical Abstract:**

**Figure:**
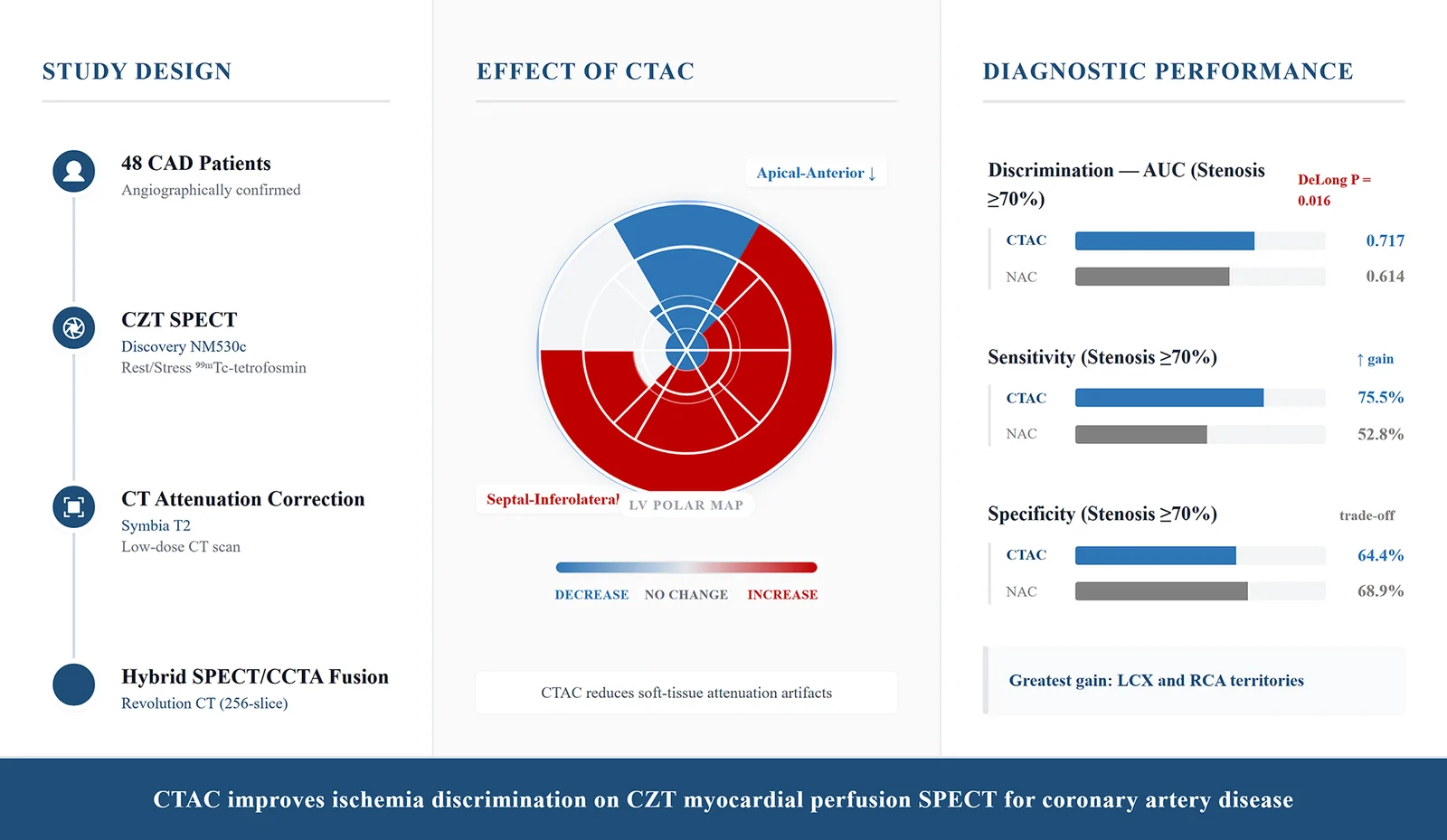

**Supplementary Information:**

The online version contains supplementary material available at https://doi.org/10.1007/s10554-026-03791-w.

## Introduction

Coronary artery disease (CAD) remains the leading cause of cardiovascular morbidity and mortality worldwide [1]. Noninvasive functional imaging, including myocardial perfusion single-photon emission computed tomography (SPECT), is central to the diagnosis and risk stratification of patients with known or suspected CAD [2, 3].

Cadmium–zinc–telluride (CZT) solid-state gamma cameras have been shown to provide superior spatial and energy resolution, as well as higher sensitivity, compared with conventional sodium iodide (NaI)–based detectors [4, 5]. These advances have enabled shorter acquisition times and lower radiation doses while maintaining diagnostic image quality [6].

Dedicated cardiac semiconductor SPECT began with cadmium telluride prototypes, including one of the earliest myocardial perfusion systems in Japan [7], before CZT became the commercial substrate. The two dedicated CZT systems share that detector material but differ fundamentally in acquisition geometry. The GE Discovery NM530c uses a stationary focused-multipinhole array, whereas the Spectrum Dynamics D-SPECT sweeps tungsten-collimated detector columns.

Despite these advantages, SPECT remains susceptible to soft-tissue attenuation artifacts that mimic or obscure perfusion defects and reduce diagnostic accuracy [8, 9]. In CZT systems these are particularly evident in the septal, inferoposterior, and inferolateral walls [4, 10]. CT attenuation correction (CTAC) compensates for these artifacts and may improve performance [11, 12]. Studies on conventional NaI SPECT show that it reduces false-positive defects [13–15], and CZT studies report improved uniformity and performance, especially inferiorly [10, 16, 17]. The value of attenuation correction is therefore established in aggregate. What remains unsettled is how far that value transfers across camera geometries, because the correction acts on an artifact distribution that the detector geometry itself creates. The stationary focal-sampling 19-pinhole array of the Discovery NM530c produces a distribution distinct from those of Anger cameras and of other CZT geometries such as the multi-detector D-SPECT, and its per-vessel CTAC performance against an angiographic reference remains sparsely characterized. This system stays in routine clinical use, so how CTAC behaves on its geometry carries direct and ongoing clinical relevance.

Hybrid cardiac SPECT/CT imaging, which integrates myocardial SPECT with coronary CT angiography (CCTA), enables precise anatomical–functional correlation between perfusion defects and culprit lesions [18–21]. It also allows myocardial segments to be assigned to coronary territories from each patient’s own coronary course rather than from a population template.

We therefore evaluated whether CT attenuation correction improves per-vessel detection of angiographically obstructive coronary territories on this platform, taking ≥ 70% diameter stenosis on the angiographic reference as the reference standard.

## Materials and methods

### Patient Population

**Fig. 1:**
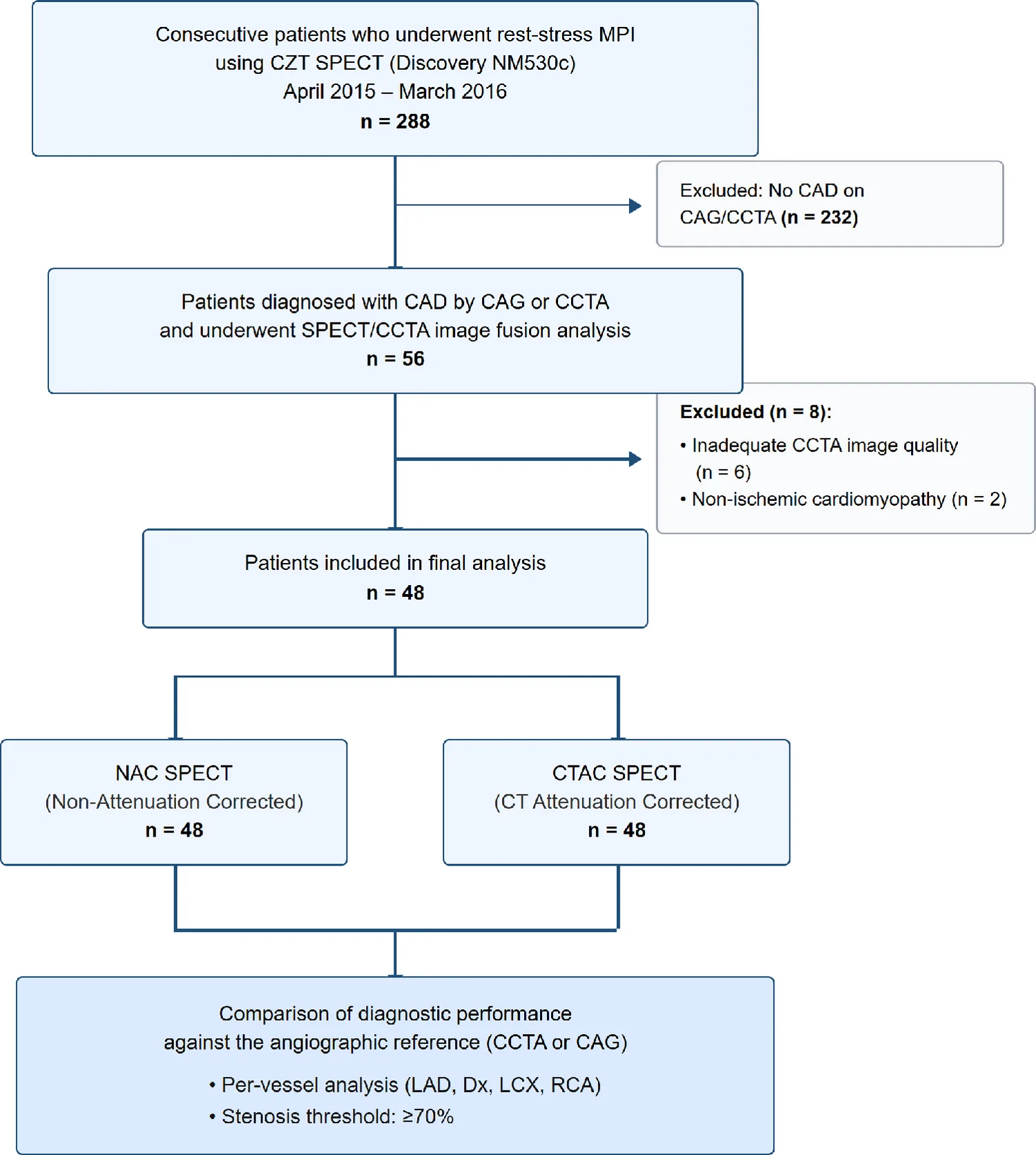
Patient selection flow diagram. Of 288 consecutive patients who underwent rest–stress myocardial perfusion SPECT using a CZT solid-state gamma camera (Discovery NM530c; GE Healthcare) between April 2015 and March 2016 for suspected CAD, 56 had angiographically confirmed CAD by CAG or CCTA and underwent hybrid cardiac SPECT/CT analysis. After excluding patients with non-ischemic cardiomyopathies (*n* = 2) and inadequate CCTA image quality (*n* = 6, comprising 3 with ECG-gating failure and 3 with motion artifact from a high heart rate), 48 patients entered the final analysis. NAC and CTAC reconstructions from the same scans were compared, with ≥ 70% diameter stenosis on the angiographic reference (CCTA or CAG) serving as the reference standard. CAD = coronary artery disease; CAG = invasive coronary angiography; CCTA = coronary CT angiography; CTAC = CT attenuation correction; CZT = cadmium–zinc–telluride; NAC = non-attenuation correction

This retrospective study included 288 consecutive patients (Fig. 1) who underwent rest–stress myocardial perfusion SPECT using a CZT solid-state gamma camera (Discovery NM530c; GE Healthcare) between April 2015 and March 2016 for suspected CAD. The initial clinical diagnosis was based on a combination of symptoms, cardiac enzymes, electrocardiographic findings, transthoracic echocardiography, and, when available, invasive coronary angiography (CAG).

The cohort median age was 74 years [IQR, 67–79] (207 men, 81 women). Of these, 56 had angiographically confirmed coronary artery disease, defined as any luminal stenosis or atherosclerotic plaque on CAG or CCTA, and underwent image fusion of myocardial perfusion SPECT and CCTA. After excluding non-ischemic cardiomyopathies (hypertrophic or dilated; *n* = 2) and inadequate CCTA image quality (*n* = 6, comprising 3 with ECG-gating failure and 3 with motion artifact from a high heart rate), 48 patients (median age 71 [IQR, 66–78]; 30 men, 18 women) with angiographically confirmed CAD entered the final analysis. No patient was excluded for inadequate SPECT-to-CT registration.

The requirement for individual informed consent was waived due to the retrospective nature of this study, and an opt-out method was used. The study protocol was approved by the Central Ethics Committee of Nippon Medical School (approval no. M-2025-496) and adhered to the principles of the Declaration of Helsinki.

## Imaging Protocols

### Myocardial Perfusion SPECT

All patients underwent rest–stress myocardial perfusion SPECT using a one-day protocol on a dedicated CZT system. At rest, 8 mCi (296 MBq) of ⁹⁹ᵐTc-tetrofosmin was injected intravenously, followed by 20 mCi (740 MBq) at stress. Stress was induced by treadmill exercise (*n* = 29; standard Bruce protocol, tracer at peak with 1 min of post-injection exercise) or adenosine (*n* = 19). Fifteen minutes after the rest injection, patients ingested milk or a lipid load and drank water to reduce subdiaphragmatic activity [10]. Rest and stress acquisitions (10 min each) began 60 and 40–50 min after injection, with longer-than-standard delays for subdiaphragmatic clearance. All acquisitions were performed in the supine position. The CZT camera employs 19 stationary detectors with multipinhole collimation focused on the cardiac region [5, 6]. Images were reconstructed by OSEM (2 iterations, 10 subsets) with a Butterworth filter (cutoff 0.52 cycles/cm, order 5) on a dedicated workstation (Xeleris; GE Healthcare; Myovation for Alcyone) [6]. Attenuation correction was the only correction applied to either reconstruction. Neither scatter correction nor motion correction was used, so the NAC-versus-CTAC comparison isolates the effect of attenuation correction alone.

Patients did not fast but abstained from caffeine for 24 h, and contraindications to pharmacologic stress were screened beforehand.

The attending physician selected treadmill exercise or adenosine stress according to each patient’s suitability. Adenosine (Adenoscan; Daiichi Sankyo) was infused at 120 µg/kg/min for 6 min (total 0.72 mg/kg), with ⁹⁹ᵐTc-tetrofosmin injected 3 min after infusion onset via a separate line.

Low-dose CT for attenuation correction used a Symbia T2 SPECT/CT scanner (Siemens Healthineers): 110 kVp, automatic tube-current modulation (CARE Dose), pitch 2, 0.8 s rotation, 5.0 mm slices, 512 × 512 matrix, reconstructed by filtered back projection with a B41 kernel [22, 23]. The CT data were used solely to generate attenuation maps, not for diagnosis. A single CT was acquired per patient and applied to both the rest and stress emission datasets. Because SPECT and CT were acquired on separate systems, patients were repositioned on the Symbia T2 on the same day, immediately after CZT SPECT. The two acquisitions shared no gantry position, so registration was performed manually in every study rather than inherited from a common frame. The SPECT and CT volumes were aligned with reference to the three left-ventricular axes, matching the cardiac axis and the rotational orientation as closely as the two acquisitions allowed, and each result was verified visually before reconstruction. The estimated effective dose of the low-dose CT was 0.55 mSv (median; range 0.36–0.93 mSv), derived from the dose-length product (median 39.2 mGy·cm) and a conversion coefficient of 0.014 mSv·mGy⁻¹·cm⁻¹ for the adult chest [24].

### Coronary CT Angiography

CCTA was performed on a 256-slice scanner (Revolution CT; GE Healthcare), with beta-blockers as needed for a heart rate < 65 bpm and sublingual nitroglycerin (0.3 mg) unless contraindicated. Retrospective ECG-gated scans used 100–120 kVp, automatic tube-current modulation (200–500 mA), 256 × 0.625 mm collimation, and 0.28–0.35 s rotation. Iodinated contrast (50–70 mL, ≥ 350 mg I/mL) was injected at 5 mL/s by bolus tracking, followed by a saline flush, and images were reconstructed at 0.625 mm thickness. The median SPECT-to-CCTA interval was 40 days [IQR, 26–54].

### Data Analysis

On a per-patient basis, each of the 17 left-ventricular segments was assigned to its coronary artery territory according to the CCTA-defined coronary course recorded as a per-segment color map. The four territories were the left anterior descending artery (LAD), the diagonal branch (Dx), the left circumflex artery (LCX), and the right coronary artery (RCA). The LAD territory comprised both the LAD-proper and the diagonal (Dx) segments. Non-attenuation-corrected (NAC) and CT attenuation-corrected (CTAC) reconstructions were generated from the same raw acquisition for every patient.

The reference standard was anatomical. A coronary territory was classified as positive when its subtending vessel reached ≥ 70% diameter stenosis on the angiographic reference, and no perfusion information entered this classification. Coronary stenosis was evaluated by CCTA or invasive coronary angiography (CAG), and in patients who underwent CAG the CAG findings were taken as the reference. On CCTA, stenosis was graded by a cardiovascular radiologist with the Coronary Artery Disease Reporting and Data System (CAD-RADS) [25] by the most severe diameter stenosis (CAD-RADS 0, 0%; 1, 1–24%; 2, 25–49%; 3, 50–69%; 4, ≥ 70%; 5, total occlusion). CAD-RADS ≥ 4 (or ≥ 70% on CAG) denoted obstructive and CAD-RADS 1–3 non-obstructive CAD, so the entry criterion of confirmed CAD (any stenosis or plaque) spanned both. Territories below the ≥ 70% threshold in CAD-confirmed patients are valid true negatives, not non-CAD.

SPECT images were interpreted independently for NAC and CTAC by two board-certified nuclear medicine physicians blinded to the angiographic reference, with discrepancies resolved by consensus. Every vessel-territory read was forced to a binary classification with no indeterminate category.

Hybrid cardiac SPECT/CT fusion images were generated by dedicated software co-registering the SPECT and CCTA datasets by anatomical landmarks, with alignment verified visually and adjusted manually when necessary. Fusion served two purposes: the per-patient assignment of myocardial segments to coronary territories and the anatomical localization of culprit lesions in the illustrative cases. It did not enter the reference standard, which rested on the angiographic finding alone.

On the 17-segment left-ventricular model [26], each segment’s perfusion defect, defined as the count reduction relative to the region of maximum myocardial uptake, was categorized in 25% increments (0%, 25%, 50%, 75%, or 100% defect) on each NAC and CTAC image set. The territory defect rate was the mean percent defect across that territory’s segments for the stress and rest phases, and the reversible defect rate was their difference, analyzed as a continuous variable rather than dichotomized.

A quantitative segmental analysis of the per-segment mean percentage uptake (100 − percent defect), comparing CTAC with NAC, was also performed, focusing on the inferior and inferolateral walls commonly affected by attenuation artifacts.

### Statistical Analysis

Continuous variables were expressed as medians with interquartile ranges [IQR]. Normality was assessed using the Shapiro-Wilk test, which confirmed a non-normal distribution of the perfusion data. The Wilcoxon signed-rank test was used to compare paired stress, rest, and reversible defect rates between NAC and CTAC reconstructions.

The pre-specified primary analysis compared per-vessel discrimination of NAC and CTAC at the ≥ 70% threshold using the area under the receiver-operating-characteristic (ROC) curve (AUC) of the continuous reversible defect rate against the angiographic reference, with the paired DeLong test for ΔAUC. Sensitivity, specificity, predictive values, and accuracy were reported at the Youden-optimal operating point with exact (Clopper-Pearson) 95% CIs. Each of the four territories (LAD, Dx, LCX, RCA) was analyzed. The RCA was excluded for the 7 LCX-dominant patients with a hypoplastic RCA, giving a per-vessel denominator of 185.

Pre-specified secondary analyses included territory-specific comparisons of the stress, rest, and reversible defect rates, and a descriptive segmental analysis mapping the CTAC − NAC difference across the 17 American Heart Association (AHA) myocardial segments. Gated SPECT, quantitative ejection fraction, and transient ischemic dilation were not part of the pre-specified protocol, and the qualitative case descriptions (Figs. 6 and 7) are exploratory post hoc observations that were not formally tested.

Categorical variables were summarized as frequencies. A two-sided *P* < 0.05 was considered significant. Territory-specific P values (~ 20 paired tests) carry no multiplicity correction and are exploratory. The primary ΔAUC 95% CI was additionally estimated by a 1,000-iteration patient-cluster bootstrap (seed 20260418) respecting within-patient correlation, and its significance was further tested by a within-patient label-permutation test (5,000 permutations swapping the CTAC and NAC predictors within each patient). Cohen κ quantified NAC-versus-CTAC agreement of the dichotomized reads (bootstrap 95% CI), and combined-reading strategies (parallel, serial, concordance-restricted) were exploratory post hoc analyses. Quantitative analyses used Python 3.11 (statsmodels, scikit-learn; DeLong by the Sun-Xu method) and are reported in Supplementary Tables S2 and S3.

## Results

### Patient Characteristics

A total of 48 patients (30 men [62.5%]; median age 71 [IQR 66–78]) were analyzed. Fifteen (31.3%) had a prior myocardial infarction and 9 (18.8%) a prior percutaneous coronary intervention (PCI) (Table 1). At the ≥ 70% threshold of the diagnostic analysis, 17 patients (35.4%) had non-obstructive CAD (stenosis < 70% in every territory), while obstructive disease involved a single territory in 15 (31.3%), two in 12 (25.0%), three in 2 (4.2%), and all four in 2 (4.2%). Coronary dominance was right in 36 (75.0%), LCX in 7 (14.6%), and balanced in 5 (10.4%) (Table 2). The 7 LCX-dominant patients had a hypoplastic RCA (inferior wall LCX-supplied), so the analysis evaluated 185 vessels (48 × 4 − 7 absent RCA territories).

**Table 1:** Baseline Patient Characteristics

Characteristic	Value (*n* = 48)
Age, years	71 [66–78]
Age range, years	54–88
Male sex	30 (62.5)
Height, m	1.60 [1.54–1.68]
Weight, kg	63.0 [52.0–72.0]
BMI, kg/m²	23.5 [21.3–25.8]
Cardiovascular risk factors	
Hypertension	18 (37.5)
Dyslipidemia	33 (68.8)
Diabetes mellitus	27 (56.2)
Current smoker	2 (4.2)
Chronic kidney disease	8 (16.7)
Cardiac history	
Prior myocardial infarction	15 (31.3)
Prior PCI	9 (18.8)
Prior CABG	6 (12.5)
Stress method	
Exercise	29 (60.4)
Adenosine	19 (39.6)
Cardiac function	
Rest LVEF, %	68.0 [60.8–77.5]
Stress LVEF, %	65.0 [58.8–75.0]
Imaging interval	
SPECT–CCTA interval, days	40 [26–54]

Data are presented as median [interquartile range] or n (%)

BMI = body mass index; CABG = coronary artery bypass grafting; CCTA = coronary CT angiography; LVEF = left ventricular ejection fraction; PCI = percutaneous coronary intervention

**Table 2:** Coronary Artery Disease Extent and Lesion Distribution

-	*n* (%)
Coronary artery dominance	
RCA dominant	36 (75.0)
LCX dominant	7 (14.6)
Balanced dominance	5 (10.4)
Extent of obstructive (≥ 70%) coronary disease	
Non-obstructive CAD (< 70% stenosis)	17 (35.4)
Single-territory disease	15 (31.3)
LAD	3 (6.3)
Dx	3 (6.3)
LCX	6 (12.5)
RCA	3 (6.3)
Two-territory disease	12 (25.0)
LAD and Dx	2 (4.2)
LAD and LCX	3 (6.3)
LAD and RCA	3 (6.3)
Dx and LCX	2 (4.2)
LCX and RCA	2 (4.2)
Three-territory disease	2 (4.2)
LAD, Dx, and LCX	2 (4.2)
Four-territory disease	2 (4.2)

Obstructive disease is defined as ≥ 70% luminal stenosis on the angiographic reference (CCTA or CAG, with CAG prioritized when performed), matching the threshold of the per-vessel diagnostic analysis (Methods). All 48 patients had coronary artery disease; the 17 patients classified as non-obstructive had angiographic stenosis < 70% in every territory. Disease extent is tabulated across the four analyzed territories (LAD, Dx, LCX, RCA); the RCA territory is anatomically absent in the 7 LCX-dominant patients with a hypoplastic RCA, in whom the inferior wall is supplied by the LCX

Dx = diagonal branch; LAD = left anterior descending artery; LCX = left circumflex artery; RCA = right coronary artery

### Comparison of Global and Regional Defect Rates (% Defect)

**Fig. 2:**
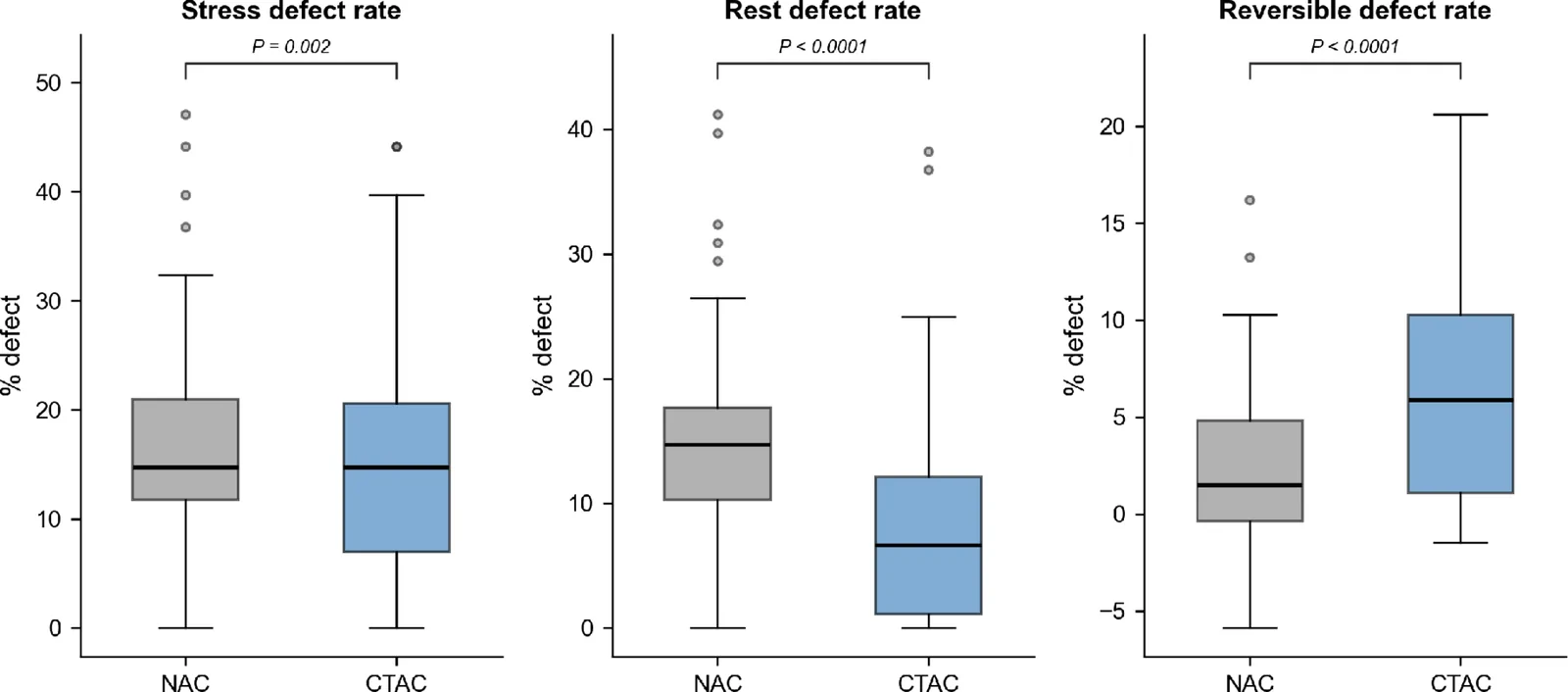
Global myocardial defect rates (% defect graded per segment in 25% increments, averaged across the 17 segments) compared between NAC and CTAC reconstructions (*n* = 48). Box plots display the median, interquartile range (box), and 1.5 × IQR whiskers, with individual data points overlaid. The stress defect rate had nearly identical medians (median 14.7% [IQR 7.0–20.6] for CTAC vs. 14.7% [11.8–21.0] for NAC; *P* = 0.002), the rest defect rate was lower with CTAC (6.6% [1.1–12.1] vs. 14.7% [10.3–17.6]; *P* < 0.0001), and the reversible defect rate (stress − rest) was higher with CTAC (5.9% [1.1–10.3] vs. 1.5% [− 0.4–4.8]; *P* < 0.0001) by Wilcoxon signed-rank test. The higher reversible defect rate with CTAC indicates improved detection of reversible perfusion defects. CTAC = CT attenuation correction; IQR = interquartile range; NAC = non-attenuation correction

The global stress defect rate had nearly identical medians between reconstructions (median 14.7% [IQR 7.0–20.6] for CTAC vs. 14.7% [11.8–21.0] for NAC; *P* = 0.002), whereas the global rest defect rate was markedly lower with CTAC (6.6% [1.1–12.1] vs. 14.7% [10.3–17.6]; *P* < 0.0001; Fig. 2). Consequently, the reversible defect rate (stress − rest) was higher with CTAC (5.9% [1.1–10.3] vs. 1.5% [− 0.4–4.8]; *P* < 0.0001; Fig. 2), indicating greater detection of reversible perfusion defects after attenuation correction.

Regional analyses, with each segment assigned to its coronary territory by the per-patient CCTA-based color map (the LAD territory including its diagonal branch), confirmed a higher reversible defect rate with CTAC in every territory: LAD (median 6.2% [0.0–12.5] vs. 3.1% [0.0–6.2]; *P* < 0.0001), Dx (8.3% [0.0–16.7] vs. 0.0% [0.0–8.3]; *P* = 0.001), LCX (0.0% [0.0–10.0] vs. 0.0% [0.0–5.0]; *P* = 0.004), and RCA (0.0% [0.0–12.5] vs. 0.0% [− 6.2–0.0]; *P* = 0.0009; Supplementary Figure S1). The reductions in stress and rest defect rates were most pronounced in the RCA territory (rest 0.0% [0.0–12.5] with CTAC vs. 31.2% [18.8–37.5] with NAC; *P* < 0.0001), reflecting effective artifact reduction in the inferior and inferolateral walls.

### Diagnostic Performance Comparison

**Fig. 3:**
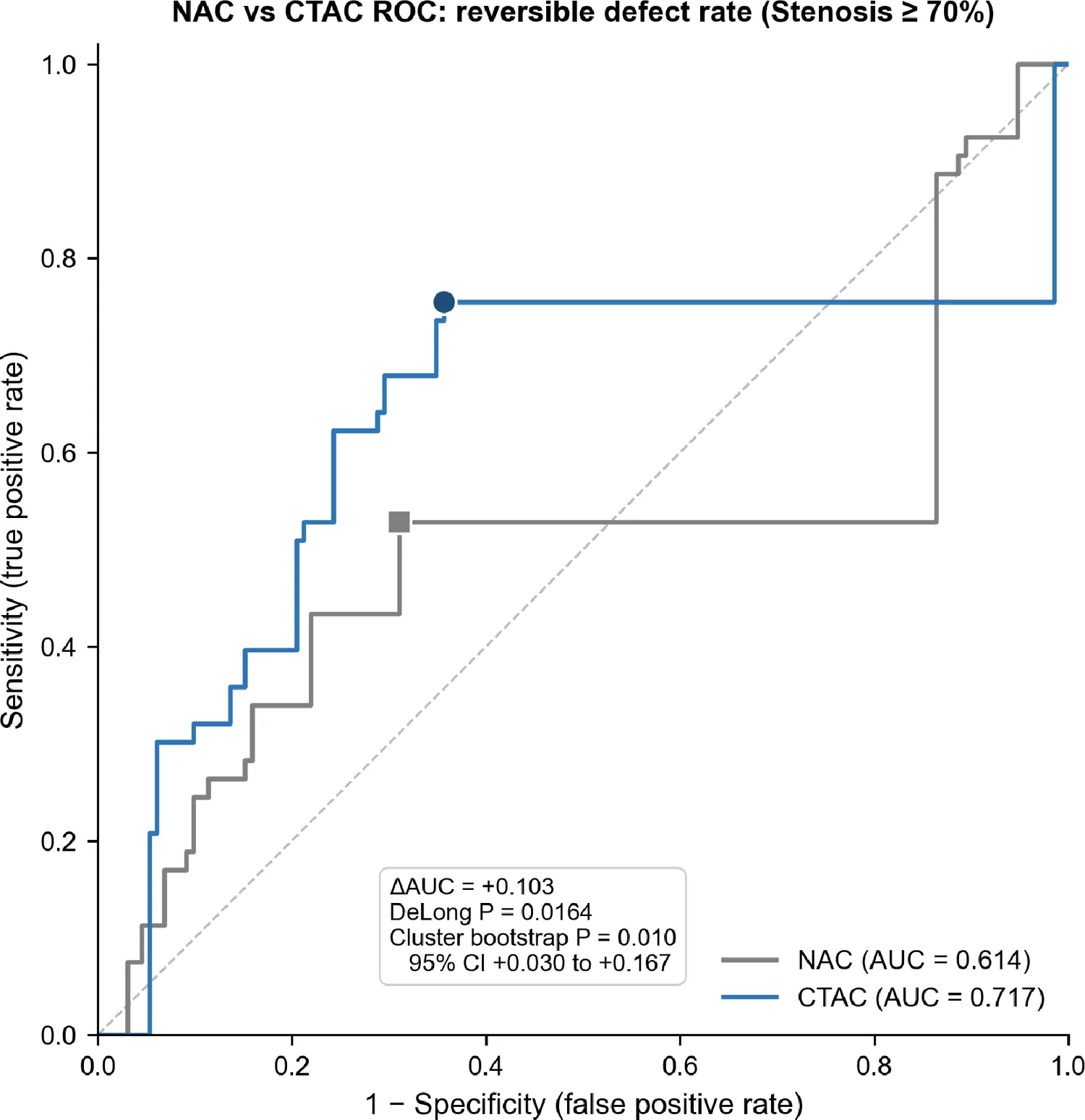
Per-vessel ROC curves of the continuous reversible defect rate (territory stress − rest % defect) for detecting CAD defined as ≥ 70% stenosis on the angiographic reference (CCTA or CAG), comparing CTAC (blue) and NAC (gray) reconstructions on the pooled vessel set (*n* = 185). CTAC discriminated better than NAC (AUC 0.717 vs. 0.614; ΔAUC 0.103, 95% CI 0.019 to 0.188; DeLong *P* = 0.0164; patient-cluster bootstrap 95% CI 0.030 to 0.167, *P* = 0.010; within-patient permutation *P* = 0.0474). Markers indicate each curve’s Youden-optimal operating point (the cutpoint that maximizes J = sensitivity + specificity − 1). Other abbreviations as in Fig. 1

On a per-vessel basis at ≥ 70% stenosis, the reversible defect rate (territory stress − rest % defect) discriminated angiographically obstructive territories significantly better with CTAC than with NAC, with an AUC of 0.717 versus 0.614 (ΔAUC 0.103, 95% CI 0.019 to 0.188; DeLong *P* = 0.0164; Fig. 3). A pre-specified patient-cluster bootstrap (1,000 iterations) was concordant (95% CI 0.030 to 0.167; *P* = 0.010), and a more conservative within-patient permutation test was also significant (*P* = 0.0474; Supplementary Table S2). At the Youden operating point, CTAC raised sensitivity to 75.5% (95% CI 61.7–86.2) from 52.8% (38.6–66.7), with a reciprocal specificity of 64.4% (55.6–72.5) versus 68.9% (60.3–76.7), primarily converting false negatives to true positives (Supplementary Table S2).

**Fig. 4:**
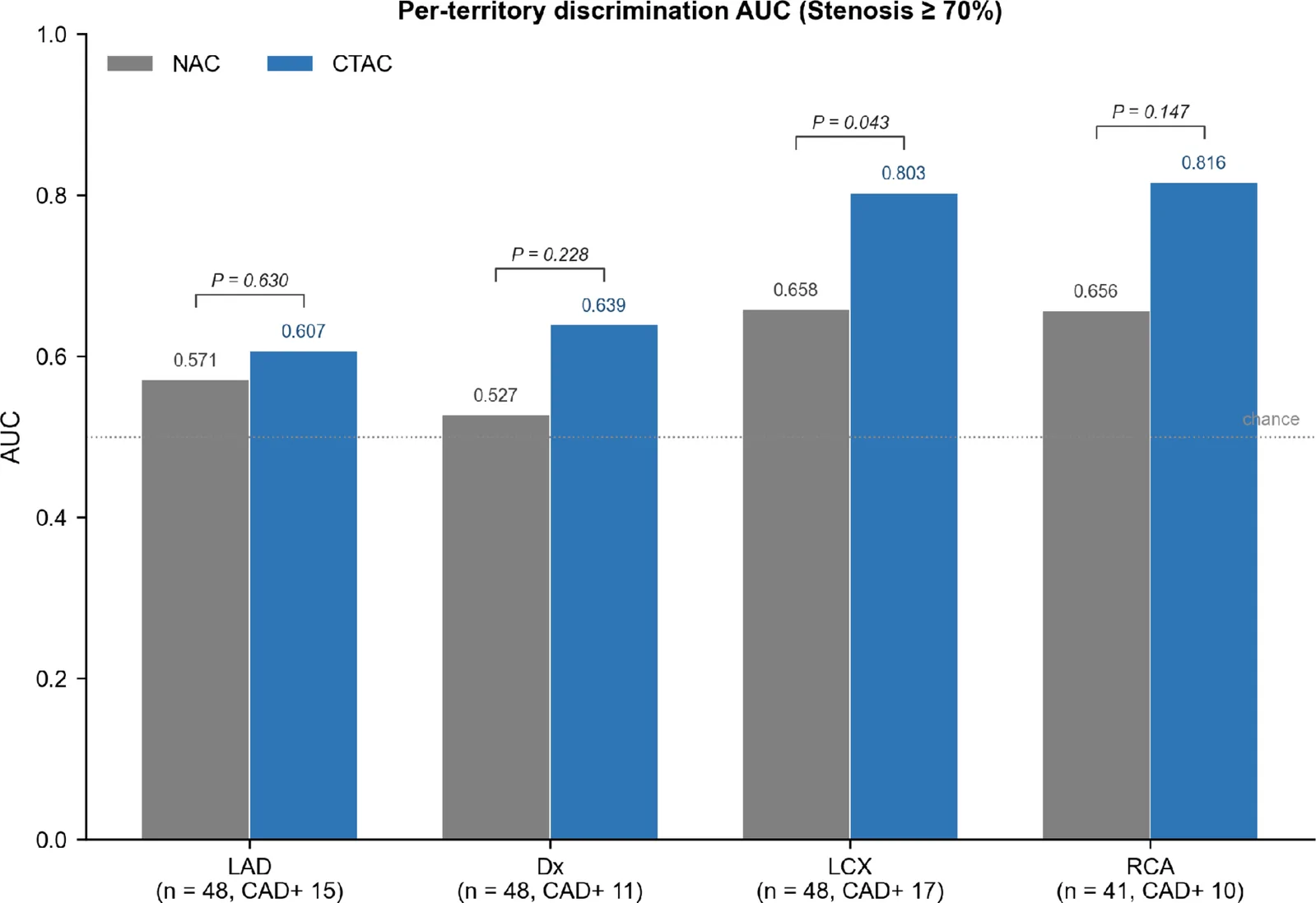
Per-territory discrimination of the reversible defect rate for detecting CAD defined as ≥ 70% stenosis on the angiographic reference (CCTA or CAG). Paired bars present the area under the ROC curve for NAC (gray) and CTAC (blue) in each coronary territory, with the paired DeLong P value. The discrimination gain with CTAC reached significance in the LCX (AUC 0.803 vs. 0.658; *P* = 0.043), with a large but non-significant gain in the RCA (0.816 vs. 0.656; *P* = 0.147), a smaller gain in the Dx (0.639 vs. 0.527), and a minimal gain in the LAD (0.607 vs. 0.571). The LAD territory comprised the LAD-proper and diagonal segments. Dx = diagonal branch; LAD = left anterior descending artery; LCX = left circumflex artery; RCA = right coronary artery. Other abbreviations as in Figs. 1 and 3

The discrimination gain was greatest in the LCX and RCA territories. The per-territory AUC for CTAC versus NAC was 0.803 versus 0.658 in the LCX (ΔAUC 0.144; DeLong *P* = 0.043) and 0.816 versus 0.656 in the RCA (ΔAUC 0.160; *P* = 0.147), with smaller, non-significant gains in the LAD (0.607 vs. 0.571) and Dx (0.639 vs. 0.527) territories (Fig. 4; Supplementary Table S1). An exploratory post hoc evaluation of combined NAC + CTAC reading strategies is reported in Supplementary Table S3.

### Segmental CTAC − NAC Differences on the 17-Segment Model

**Fig. 5:**
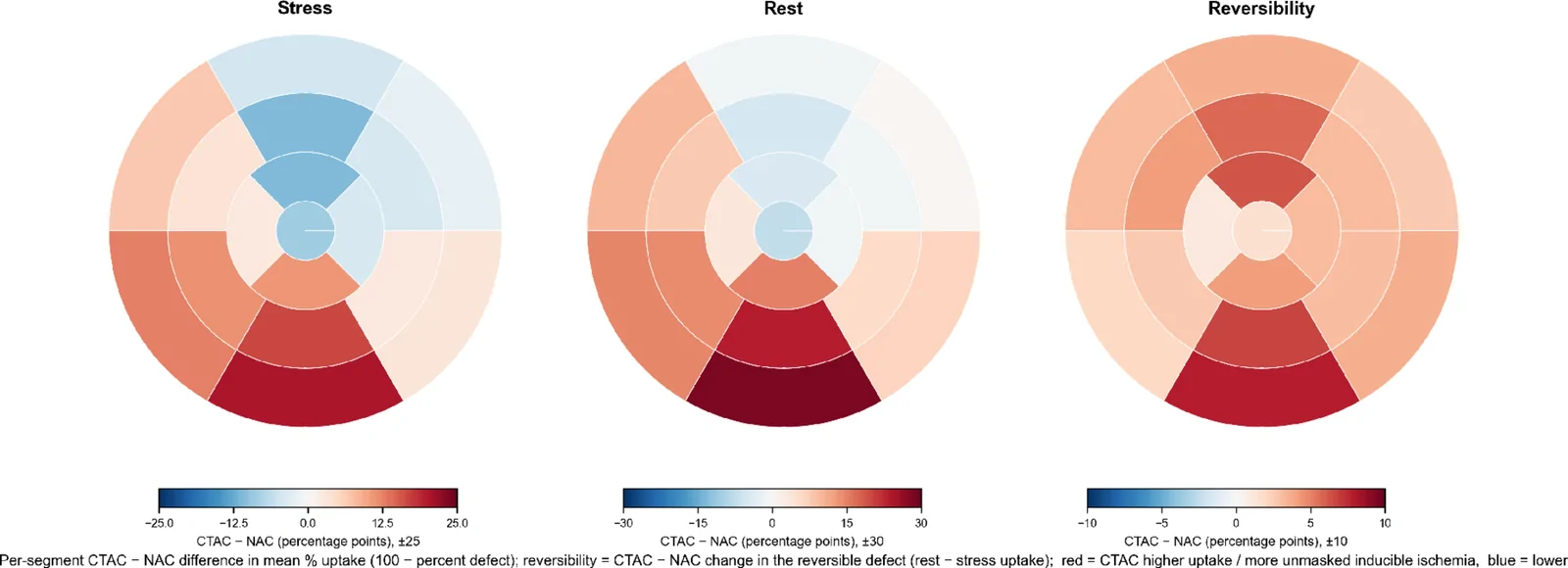
Segmental CTAC − NAC differences in mean percentage uptake on the 17-segment AHA model (*n* = 48). Three polar maps display, for each segment, the CTAC − NAC difference in the mean percentage uptake (100 − percent defect) for the stress and rest phases and the CTAC − NAC change in the reversible defect (rest − stress uptake) for the reversibility panel, in standard AHA orientation (anterior at the top, septum at the left, inferior at the bottom, lateral at the right). For the stress and rest panels, red indicates that CTAC raised the percentage uptake and blue that CTAC lowered it; for the reversibility panel, red indicates a CTAC-induced increase in the reversible defect. CTAC most raised the uptake in the inferior and inferoseptal walls (basal inferior segment, + 20.3% points at stress and + 28.1 at rest). Because CTAC raised the rest uptake more than the stress uptake, the reversible defect (rest − stress uptake) rose in every segment (+ 1.0 to + 7.8% points; red), reflecting recovery of reversible defects that attenuation had suppressed. Each panel uses its own symmetric diverging color scale (stress ± 25, rest ± 30, reversibility ± 10% points) so that the smaller-magnitude reversibility differences remain legible. AHA = American Heart Association; CTAC = CT attenuation correction; NAC = non-attenuation correction

On the 17-segment AHA model, CTAC raised the stress and rest uptake (100 − percent defect; *n* = 48) predominantly in the inferior and inferoseptal walls, with the largest increases in the basal inferior segment (+ 20.3% points at stress and + 28.1 at rest), while modestly lowering it anteriorly and apically. Because CTAC raised the rest uptake more than the stress uptake, the reversible defect rose in every segment (+ 1.0 to + 7.8% points), most in the basal and mid inferior segments (Fig. 5).

### Case Presentations

**Fig. 6:**
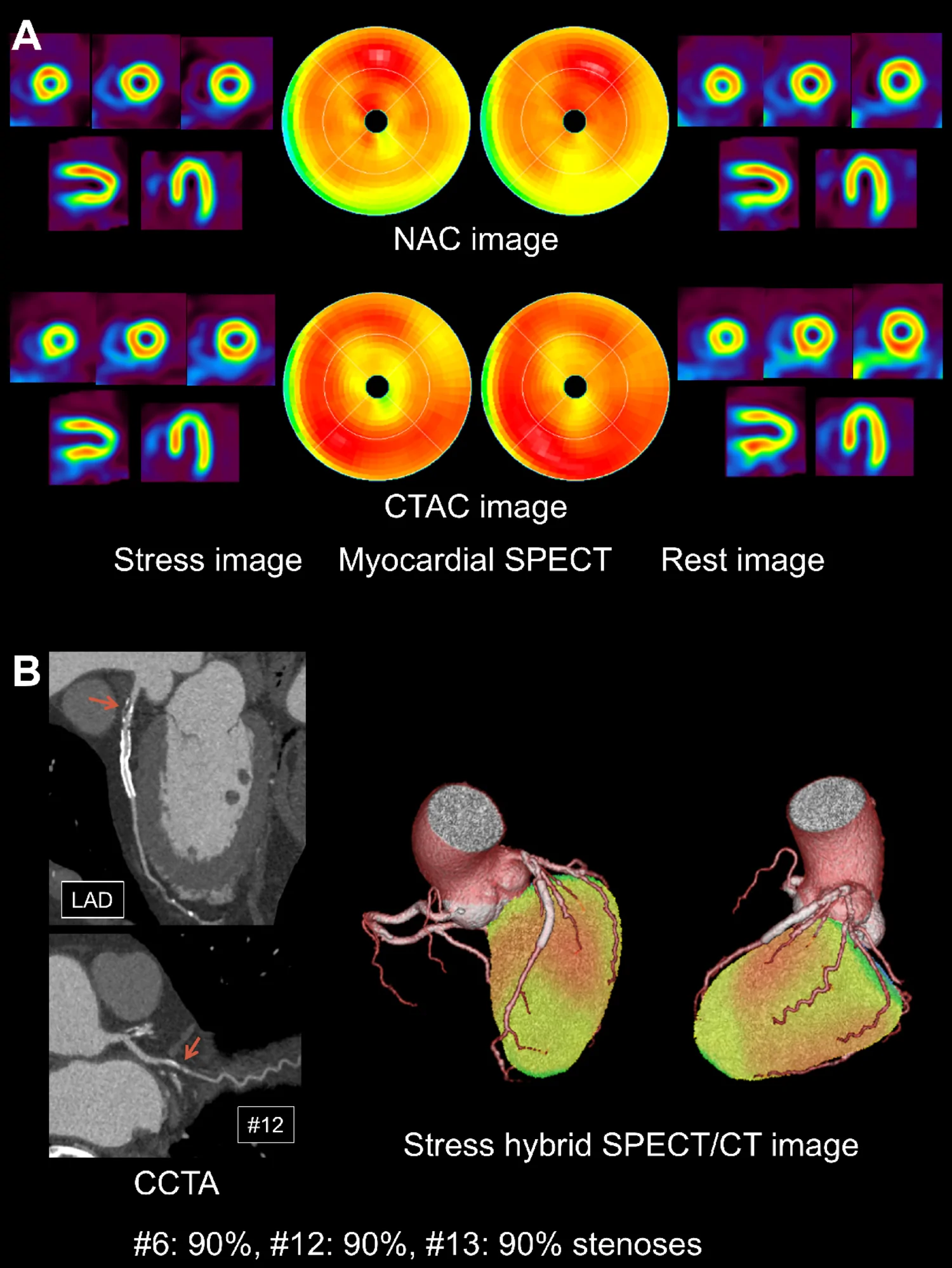
Representative case of myocardial ischemia in the LAD and LCX territories. **(A)** Rest–stress myocardial perfusion SPECT. Polar maps (center) are flanked by short-axis SPECT slices for the stress (left) and rest (right) studies. The top half shows the NAC reconstructions and the bottom half the CTAC reconstructions. CTAC reveals clearer perfusion defects in the apical inferior wall and basal anterolateral wall. **(B)** Hybrid cardiac SPECT/CT imaging of the same patient. Arrows indicate the culprit lesions, severe proximal stenoses of the LAD and its branches (#6: 90%, #12: 90%, #13: 90%) corresponding anatomically to the perfusion defects in Panel A. Coronary segment numbers (#6, #12, #13) follow the AHA coronary segmentation scheme. Abbreviations as in Figs. 1 and 4

**Fig. 7:**
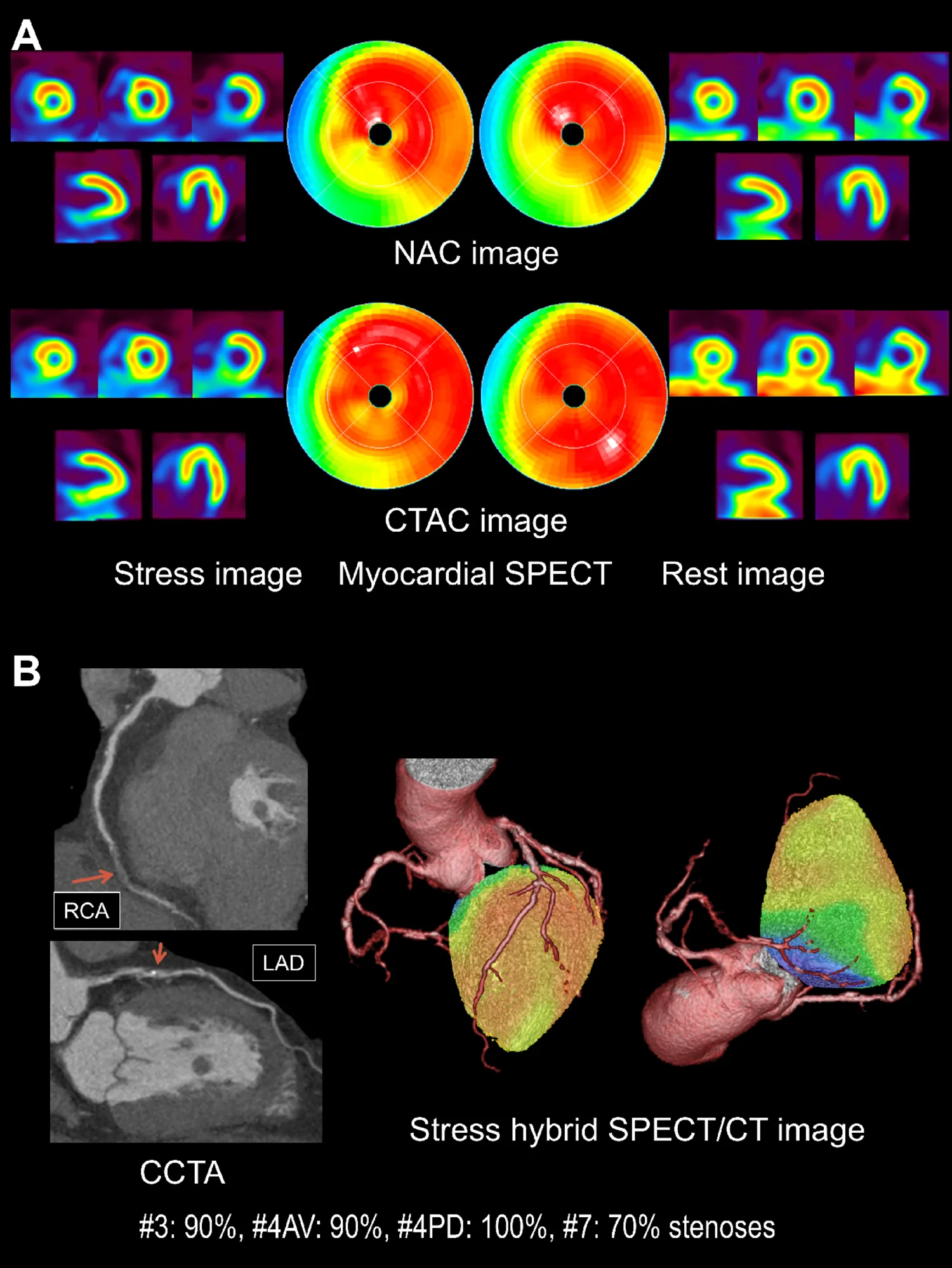
Representative case of myocardial ischemia in the RCA territory. **(A)** Rest–stress myocardial perfusion SPECT, layout as in Fig. 6A. CTAC reveals more distinct perfusion defects in the inferior wall. **(B)** Hybrid cardiac SPECT/CT imaging of the same patient. Arrows indicate the culprit RCA lesions (#3: 90%, #4AV: 90%, #4PD: 100%) that correspond anatomically to the inferior-wall defects in Panel A. A concomitant moderate stenosis in LAD segment #7 (70%) was not associated with inducible ischemia. Coronary segment numbers (#3, #4AV, #4PD, #7) follow the AHA coronary segmentation scheme. Abbreviations as in Figs. 1 and 4

Two representative cases illustrate the pattern. In the first (Fig. 6), CTAC clarified defects in the LAD and LCX territories that corresponded on fused images to severe stenoses of segments #6, #12, and #13. In the second (Fig. 7), CTAC clarified an RCA-territory defect corresponding to stenoses of #3, #4AV, and #4PD, whereas a moderate LAD #7 stenosis showed no inducible defect.

## Discussion

This study evaluated the diagnostic impact of CTAC on CZT myocardial perfusion SPECT against an angiographic reference standard. CTAC significantly improves per-vessel discrimination of angiographically obstructive territories on the stationary 19-pinhole geometry. With the reversible defect rate analyzed as a continuous variable, the per-vessel AUC rose from 0.614 with NAC to 0.717 with CTAC (ΔAUC 0.103; DeLong *P* = 0.0164), corroborated by a patient-cluster bootstrap (*P* = 0.010) and a more conservative within-patient permutation test (*P* = 0.0474). The gain is carried by sensitivity rather than specificity, reversing the direction reported for Anger-camera systems and defining the platform-specific contribution of this work.

### Improvement in Diagnostic Sensitivity

At the Youden-optimal operating point, sensitivity rose to 75.5% with CTAC from 52.8% with NAC. In conventional SPECT, the meta-analysis by Huang et al. [27] found that attenuation correction primarily improved specificity (pooled 68% to 83%) without changing sensitivity (80% in both arms), by reducing false-positive defects. A subsequent meta-analysis from the same group analyzed the diagnostic effect of attenuation correction separately for each coronary artery [28]. Our CZT study showed the opposite direction, with CTAC primarily improving sensitivity, likely reflecting the attenuation distribution of the focal-sampling 19-pinhole NM530c geometry. Whereas conventional parallel-hole collimation produces prominent inferior artifacts that mimic defects (reducing specificity), the NM530c produces more diffuse inferoposterior and inferolateral attenuation that obscures true defects (reducing sensitivity) [4, 8]. Oddstig et al. [29] confirmed this, showing that the NM530c attenuation artifact is displaced laterally and is larger in extent than on a conventional camera.

Herzog et al. [16] validated CTAC on CZT SPECT (*n* = 66) with high segmental correlation and > 96% clinical agreement, and Caobelli et al. [17] showed that co-registered external CT improved CZT diagnostic performance, especially for the RCA and overall specificity. Our findings extend these to per-vessel discrimination on the stationary 19-pinhole geometry, with myocardial segments assigned to territories from each patient’s own coronary anatomy and the reversible defect rate analyzed as a continuous variable. The contribution is incremental in concept and platform-specific in substance, addressing how an established correction behaves on a geometry whose artifact distribution differs from those of the systems in which it was validated. The Discovery NM530c’s CAD diagnostic performance is well established in a 1,000-patient multicenter registry [30] and a recent meta-analysis (pooled sensitivity 83.5%, specificity 75.8%) [31].

### Reduction of Attenuation Artifacts

The decrease in stress and rest defect rates with CTAC reflects effective reduction of soft-tissue attenuation artifacts in the inferoposterior and lateral segments, while the concomitant rise in the reversible defect rate indicates that the correction recovered reversible defects that attenuation had previously obscured. This follows from an asymmetric effect on the two phases. CTAC lowered the rest defect rate substantially (median 14.7% to 6.6%) while leaving the stress defect rate essentially unchanged (14.7%), widening the stress–rest gradient. In reversibly ischemic territories, resting perfusion is normal, so the resting defect was attributable to attenuation alone and was largely abolished by CTAC, whereas on stress images, superimposed true ischemia limited count recovery. These polar-map changes are consistent with the known distribution of attenuation artifacts in CZT SPECT [8–10] and with Sharma et al. [15], who reported significantly smaller inferior defects with CTAC. On this platform, the reduction reflects two camera-specific sources: soft-tissue attenuation artifacts, which CTAC reduced significantly, and the multiplexing streak artifacts intrinsic to the stationary 19-pinhole, focal-sampling geometry of the Discovery NM530c, in which pinhole sampling and the CZT low-energy tail also complicate scatter correction [32]. Because both arise from this camera’s detector structure, the CTAC benefit is specific to the NM530c and should not be extrapolated to Anger-camera systems, to the multi-detector D-SPECT, or to newer integrated CZT SPECT/CT scanners.

A secondary source is high subdiaphragmatic activity (liver, stomach, bowel), which contaminates the inferior wall in two opposite ways [33, 34]. Spatial overlap and scatter artifactually increase counts in adjacent segments [9], whereas from a distance the same activity produces multiplexing streaks that reduce counts and mimic defects. CTAC amplifies the abdominal activity itself yet relatively attenuates both artifacts, reducing their net contamination. This matches prior CZT observations of hepatic and bowel activity degrading inferior-wall quality [10], though it was not directly quantified here.

The SPAG multicenter study by Genovesi et al. [14] showed that both CTAC and gated SPECT improve performance, with CTAC particularly beneficial in overweight patients and for RCA disease.

### Trade-Off Between Sensitivity Gain and Specificity Loss

The principal downside of CTAC is a modest specificity reduction (overall 64.4% vs. 68.9% with NAC at the Youden point), reflecting additional false-positive territories, with the smallest per-territory gain in the LAD (Fig. 4, Supplementary Table S1). A false-positive defect in the large LAD territory can prompt angiography or revascularization, so this trade-off is clinically relevant. Hybrid cardiac SPECT/CT imaging mitigates it, as the anatomical channel lets the reader verify whether a CTAC-induced defect colocalizes with a stenosis and downgrade discordant findings as residual artifact.

### Clinical Implications of Hybrid Cardiac SPECT/CT Imaging

Hybrid cardiac SPECT/CT imaging localizes the culprit lesion by combining functional and anatomical information, an advantage over CCTA or CAG alone [18, 19, 35]. Pazhenkottil et al. showed that CTAC improved the prognostic value of perfusion imaging [36] and that hybrid SPECT/CT influenced treatment strategy [37]. Our cases (Figs. 6 and 7) illustrate this localization of lesions missed on NAC.

Ito et al. [10] found that both CTAC and prone imaging reduced attenuation artifacts in CZT SPECT and improved specificity, with CTAC providing higher overall accuracy. Concordantly, our data show that CTAC complements the specificity gain of prone imaging by improving sensitivity.

Whether CTAC should be routine in CZT SPECT has been debated [38, 39]. Koopman et al. [38] noted increased operator variability with CTAC processing, supporting standardized protocols. Nevertheless, recent American Society of Nuclear Cardiology (ASNC) [40] and European Association of Nuclear Medicine (EANM) [26] guidelines recommend CTAC when available.

In a post hoc exploration of combined reading strategies (Supplementary Table S3), neither a parallel nor a serial rule uniformly outperformed CTAC alone, and agreement between dichotomized NAC and CTAC reads was moderate (Cohen κ = 0.561). The discordant vessels may be the most clinically uncertain and stand to benefit most from hybrid adjudication.

### Limitations

The retrospective single-center design and small sample size (*n* = 48) limit the precision of the per-territory estimates. No a priori sample-size calculation was performed, as the study was a consecutive-series diagnostic-accuracy evaluation. The 185 per-vessel observations yield 95% CIs of roughly ± 10% points overall and ± 15–30 points for individual territories (Supplementary Table S1). Because inclusion required angiographic confirmation of CAD, disease-negative participants were excluded by design. This verification and spectrum bias means that specificity derives only from non-diseased territories within CAD-positive patients, and that the cohort does not represent the routine population with suspected CAD in whom myocardial perfusion SPECT is ordinarily requested. These estimates should not be extrapolated to lower-prevalence populations. As CAG was not performed in every patient, the anatomical reference relied on CCTA grading, so residual CCTA–CAG discordance cannot be excluded.

The reference standard was anatomical. A ≥ 70% diameter stenosis does not invariably produce hemodynamically significant ischemia, and no physiological confirmation by invasive fractional flow reserve, instantaneous wave-free ratio, CT-derived fractional flow reserve, or PET myocardial blood flow was available. What this study measures is therefore the detection of angiographically obstructive territories rather than of physiologically verified ischemia, and the reported discrimination should be read in those terms.

The per-vessel analysis treated each territory as independent. The patient-cluster bootstrap (Supplementary Table S2) partially mitigates this but does not replace a per-patient endpoint. Inter-reader agreement was not calculated because discrepancies were resolved by consensus. The κ in Supplementary Table S3 quantifies NAC-versus-CTAC agreement of the consensus reads. Territory-specific P values are exploratory (no multiplicity correction across the ~ 20 paired tests). Sex-stratified analysis was not performed. With 30 men and 18 women, the cohort was underpowered for sex-specific attenuation patterns (diaphragmatic in men, breast in women).

The proposed sensitivity-driven mechanism on the Discovery NM530c, diffuse attenuation across the inferoposterior and inferolateral walls obscuring true defects, is a hypothesis consistent with our polar-map findings (Fig. 5) and prior CZT reports [4, 8, 10] but not validated by phantom studies on this geometry. These results are specific to this stationary 19-pinhole system and should not be assumed to hold for other CZT camera geometries or for newer integrated CZT SPECT/CT scanners, whose detector configurations and attenuation characteristics differ. CTAC was performed on a separate SPECT/CT, so repositioning between the two acquisitions could introduce misregistration [39]. Every study therefore required manual registration against the three left-ventricular axes, and no patient was excluded for inadequate alignment. Residual registration error nevertheless cannot be excluded, and an integrated system would remove this step altogether. The 40-day [IQR, 26–54] SPECT-to-CCTA interval may add registration and biological variability despite per-case coregistration. The Youden-point sensitivity and specificity are in-sample and therefore optimistic, requiring external validation. Gated SPECT, ejection fraction, and transient ischemic dilation were not compiled, precluding a CTAC-versus-gating comparison [14], and with only 2 events over 5.0 years the cohort was underpowered for prognosis. Larger prospective studies including CAD-negative participants are needed to determine whether CTAC-guided management improves outcomes.

### Future Directions

Integrated CZT SPECT/CT scanners with built-in CT would remove separate-system misregistration, and deep learning–based attenuation correction that synthesizes CT maps from emission data may eliminate the additional CT acquisition and its radiation altogether [41].

### New Knowledge Gained and Clinical Implications

This study characterizes platform-specific CTAC performance against an angiographic reference in 185 per-vessel observations on the focal-sampling 19-pinhole Discovery NM530c, whose attenuation-artifact regime differs from those of Anger cameras and other CZT geometries. On this platform, CTAC improves discrimination by recovering defects obscured by diffuse attenuation rather than by eliminating false positives (per-vessel AUC 0.717 vs. 0.614; DeLong *P* = 0.0164). The gain is territory-specific, greatest in the LCX and RCA, with a modest specificity reduction that hybrid imaging is positioned to adjudicate. The 2024 ESC guidelines for chronic coronary syndromes place functional imaging and hybrid anatomical–functional strategies at the center of the diagnostic pathway [2], and CTAC with hybrid cardiac SPECT/CT imaging serves that pathway in suspected multivessel disease, both for guideline-directed evaluation [42] and for culprit-lesion localization ahead of revascularization decisions [43–45].

## Conclusions

In patients with angiographically confirmed CAD, CTAC significantly improves the per-vessel discrimination of angiographically obstructive territories on CZT myocardial perfusion SPECT (AUC 0.717 vs. 0.614; ΔAUC 0.103; DeLong *P* = 0.0164), most prominently in the LCX and RCA. The benefit is carried by sensitivity, through recovery of defects obscured by soft-tissue attenuation, at a modest cost in specificity. This sensitivity-led direction is specific to the stationary 19-pinhole geometry and reverses the specificity-led benefit reported for Anger cameras, which argues for platform-specific evaluation whenever attenuation correction is introduced into a CZT workflow.

## Supplementary Information

Below is the link to the electronic supplementary material.

Supplementary Material 1

Supplementary Material 2

Supplementary Material 3

Supplementary Material 4

## Data Availability

The data are available from the corresponding author on reasonable request.

## Abbreviations

CAD: coronary artery disease
CCTA: coronary CT angiography
CTAC: CT attenuation correction
CZT: cadmium–zinc–telluride
LAD: left anterior descending artery
LCX: left circumflex artery
NAC: non-attenuation correction
RCA: right coronary artery
SPECT: single photon emission computed tomography
