# Supplementary material for: Platform-specific impact of CT attenuation correction on CZT myocardial perfusion SPECT: per-vessel detection of obstructive coronary artery disease
Source: Int J Cardiovasc Imaging. 2026 Aug 12;42(11):2217–31. doi: 10.1007/s10554-026-03791-w (PMC13745409; doi:10.1007/s10554-026-03791-w)
Supplement: Supplementary file 1 [file 10554_2026_3791_MOESM2_ESM.docx]

**Supplementary Table S1. Per-Territory Discrimination (AUC) of the Reversible Defect Rate at the ≥ 70% Stenosis Threshold**

| **Territory** | **Vessels (n)** | **CAD+ (n)** | **AUC CTAC** | **AUC NAC** | **ΔAUC** | **DeLong P** |
| --- | --- | --- | --- | --- | --- | --- |
| LAD (incl. Dx) | 48 | 15 | 0.607 | 0.571 | 0.036 | 0.630 |
| Dx | 48 | 11 | 0.639 | 0.527 | 0.112 | 0.228 |
| LCX | 48 | 17 | 0.803 | 0.658 | 0.144 | **0.043** |
| RCA | 41 | 10 | 0.816 | 0.656 | 0.160 | 0.147 |
| **Overall (pooled)** | **185** | **53** | **0.717** | **0.614** | **0.103** | **0.0164** |

AUC = area under the receiver-operating-characteristic curve of the continuous reversible defect rate (territory stress − rest % defect, where the % defect is graded per segment in 25% increments and averaged across the territory's segments) against the ≥ 70% angiographic reference. ΔAUC = CTAC − NAC, compared by the paired DeLong test (Sun–Xu method). For the pooled analysis the ΔAUC 95% CI was 0.019 to 0.188 (analytic) and 0.030 to 0.167 by a 1,000-iteration patient-cluster bootstrap (P = 0.010). At the Youden-optimal operating point the pooled sensitivity/specificity were 75.5% (95% CI 61.7–86.2) / 64.4% (55.6–72.5) for CTAC and 52.8% (38.6–66.7) / 68.9% (60.3–76.7) for NAC. The per-vessel denominator is 185 = (48 patients × 4 territories) − 7 anatomically absent RCA territories in patients with LCX-dominant coronary anatomy and a hypoplastic RCA. Each segment was assigned to its coronary territory on a per-patient basis from the CCTA coronary course; the LAD territory comprises the LAD-proper and diagonal (Dx) segments, and the separate Dx row is the diagonal subset. Bold P values indicate statistical significance (P < 0.05).

AUC = area under the curve; CAD = coronary artery disease; CCTA = coronary CT angiography; CTAC = CT attenuation correction; Dx = diagonal branch; LAD = left anterior descending artery; LCX = left circumflex artery; NAC = non-attenuation correction; RCA = right coronary artery.
