# Supplementary material for: Platform-specific impact of CT attenuation correction on CZT myocardial perfusion SPECT: per-vessel detection of obstructive coronary artery disease
Source: Int J Cardiovasc Imaging. 2026 Aug 12;42(11):2217–31. doi: 10.1007/s10554-026-03791-w (PMC13745409; doi:10.1007/s10554-026-03791-w)
Supplement: Supplementary file 2 [file 10554_2026_3791_MOESM3_ESM.docx]

**Supplementary Table S2. Per-Vessel Discrimination of the Reversible Defect Rate: DeLong Paired ROC Test, Bootstrap, and Operating Points (≥ 70% threshold)**

**A. ROC discrimination (pooled, n = 185 vessels)**

| **Quantity** | **AUC / ΔAUC** | **95% CI** |
| --- | --- | --- |
| AUC CTAC | 0.717 | — |
| AUC NAC | 0.614 | — |
| **ΔAUC (CTAC − NAC), analytic (Sun–Xu DeLong)** | **+0.103** | **+0.019 to +0.188** |
| **ΔAUC (CTAC − NAC), patient-cluster bootstrap (n_boot = 1000, 48 clusters)** | **+0.103** | **+0.030 to +0.167** |

Analytic DeLong test: z = 2.40; P = 0.0164. Patient-cluster bootstrap (resampling 48 patient clusters with replacement; vessels nested within patient; random seed 20260418): two-sided percentile P = 0.010. Within-patient label-permutation test (5,000 permutations swapping the CTAC and NAC predictors within each patient under the null of modality exchangeability): two-sided P = 0.0474. The predictor is the continuous reversible defect rate (territory stress − rest % defect, where the % defect is graded per segment in 25% increments and averaged across the territory's segments). The analytic DeLong test treats vessels as independent; the patient-cluster bootstrap accounts for within-patient correlation across territories. The discrimination advantage of CTAC over NAC is significant by all three tests: the DeLong test (P = 0.0164), the patient-cluster bootstrap (P = 0.010), and the most conservative within-patient permutation test (P = 0.0474).

**B. Operating points (Youden-optimal)**

| **Reconstruction** | **Cutoff (reversible defect rate, %)** | **Sensitivity (95% CI)** | **Specificity (95% CI)** |
| --- | --- | --- | --- |
| CTAC | 5.6 | 75.5% (61.7–86.2) | 64.4% (55.6–72.5) |
| NAC | 2.8 | 52.8% (38.6–66.7) | 68.9% (60.3–76.7) |

Sensitivity and specificity are reported at each curve's Youden-optimal cutpoint (maximizing sensitivity + specificity − 1); these in-sample operating characteristics are optimistic and require external validation. Exact (Clopper–Pearson) 95% CIs. The per-vessel denominator is 185 = (48 patients × 4 territories) − 7 anatomically absent RCA territories in LCX-dominant patients with a hypoplastic RCA.

AUC = area under the curve; CI = confidence interval; CTAC = CT attenuation correction; NAC = non-attenuation correction; ROC = receiver operating characteristic.
