# Supplementary material for: Platform-specific impact of CT attenuation correction on CZT myocardial perfusion SPECT: per-vessel detection of obstructive coronary artery disease
Source: Int J Cardiovasc Imaging. 2026 Aug 12;42(11):2217–31. doi: 10.1007/s10554-026-03791-w (PMC13745409; doi:10.1007/s10554-026-03791-w)
Supplement: Supplementary file 3 [file 10554_2026_3791_MOESM4_ESM.docx]

**Supplementary Table S3. Post-Hoc Evaluation of Combined NAC + CTAC Reading Strategies (≥ 70% threshold)**

Reading strategies were compared on the same per-vessel dataset used for the primary analysis, dichotomizing each reconstruction's continuous reversible defect rate at its Youden-optimal cutoff (CTAC ≥ 5.6%, NAC ≥ 2.8%). These dichotomized analyses are exploratory and, because the cutoffs are data-derived, in-sample optimistic.

| **Strategy** | **Definition** |
| --- | --- |
| NAC alone | Non-attenuation-corrected reading only |
| CTAC alone | CT attenuation-corrected reading only |
| OR (parallel) | Positive if either NAC or CTAC is positive |
| AND (serial) | Positive only if both NAC and CTAC are positive |
| Concordant-only | Restricted to vessels with matching NAC and CTAC calls |

**A. Diagnostic performance (n = 185 vessels; 40 discordant, 21.6%)**

| **Strategy** | **TP** | **FN** | **FP** | **TN** | **Sensitivity (95% CI)** | **Specificity (95% CI)** | **PPV** | **NPV** | **Accuracy** |
| --- | --- | --- | --- | --- | --- | --- | --- | --- | --- |
| NAC alone | 28 | 25 | 41 | 91 | 52.8% (38.6–66.7) | 68.9% (60.3–76.7) | 40.6% | 78.4% | 64.3% |
| CTAC alone | 40 | 13 | 47 | 85 | 75.5% (61.7–86.2) | 64.4% (55.6–72.5) | 46.0% | 86.7% | 67.6% |
| OR (parallel) | 42 | 11 | 56 | 76 | 79.2% (65.9–89.2) | 57.6% (48.7–66.1) | 42.9% | 87.4% | 63.8% |
| AND (serial) | 26 | 27 | 32 | 100 | 49.1% (35.1–63.2) | 75.8% (67.5–82.8) | 44.8% | 78.7% | 68.1% |
| Concordant-only | 26 | 11 | 32 | 76 | 70.3% (53.0–84.1) | 70.4% (60.8–78.8) | 44.8% | 87.4% | 70.3% |

The concordant-only strategy is restricted to the 145 vessels with matching NAC and CTAC calls.

**B. Agreement between NAC and CTAC reads**

| **Threshold** | **Concordant** | **Discordant** | **Percent agreement** | **Cohen κ (95% CI)** |
| --- | --- | --- | --- | --- |
| ≥ 70% | 145 | 40 | 78.4% | 0.561 (0.437–0.667) |

The Cohen κ 95% CI was derived from a 1,000-iteration patient-cluster bootstrap (random seed 20260418), which respects within-patient correlation across the four vessel territories. The lower bound exceeds zero, supporting moderate between-reconstruction agreement of the dichotomized reads.

**C. Pairwise McNemar comparisons (paired classifications)**

| **Comparison** | **b (A−, B+)** | **c (A+, B−)** | **P value** | **Method** |
| --- | --- | --- | --- | --- |
| OR vs CTAC | 0 | 11 | 0.001 | Exact binomial |
| AND vs CTAC | 29 | 0 | < 0.0001 | Continuity-corrected χ² |
| OR vs NAC | 0 | 29 | < 0.0001 | Continuity-corrected χ² |
| AND vs NAC | 11 | 0 | 0.001 | Exact binomial |

Neither the parallel OR rule nor the serial AND rule uniformly outperformed CTAC alone. The serial AND rule yielded the highest specificity (75.8%) at the cost of sensitivity, suggesting a potential rule-in adjunct, whereas the parallel OR rule maximized sensitivity (79.2%). The approximately 22% of vessels with discordant NAC and CTAC calls may represent the most clinically uncertain subset, potentially benefiting from adjudication by hybrid cardiac SPECT/CT imaging.

The per-vessel denominator is 185 = 48 patients × 4 territories − 7 anatomically absent RCA territories. The 7 absent territories correspond to the 7 patients with LCX-dominant coronary anatomy and a hypoplastic ("small") RCA, in whom the posterior descending and posterior left-ventricular branches are supplied by the LCX. These patients have no RCA-supplied myocardium to evaluate and are excluded from the analysis.

CTAC = CT attenuation correction; NAC = non-attenuation correction; NPV = negative predictive value; PPV = positive predictive value.
